# Effect of arginine-fluoride varnish on preventing enamel erosion by paediatric liquid medicaments

**DOI:** 10.1186/s12903-023-03621-8

**Published:** 2023-11-20

**Authors:** Kimberley Yip, Mohamed Mahmoud Abdalla, Mohammed Nadeem Bijle, Cynthia Yiu

**Affiliations:** 1 Paediatric Dentistry, Faculty of Dentistry, The University of Hong Kong, 34 Hospital Road, Prince Philip Dental Hospital, Sai Ying Pun, Hong Kong Island, Hong Kong; 2 Dental Biomaterials, Faculty of Dental Medicine, Al-Azhar University, Cairo, Egypt

**Keywords:** Arginine, Erosive wear, Fluoride, Paediatric liquid medicaments, Varnish

## Abstract

**Background:**

The study objective was to examine the effect of arginine-sodium fluoride (Arg-NaF) varnish on preventing enamel erosion by acidic paediatric liquid medicaments (PLM).

**Methods:**

The treatment groups were: 1) 2% Arg-NaF; 2) 4% Arg-NaF; 3) 8% Arg-NaF; 4) NaF; 5) MI (CPP-ACFP) varnishes; and 6) no varnish. The pH of PLM (paracetamol and chlorpheniramine) was measured at baseline and after immersing the Perspex® blocks coated with varnishes at 0 min, 30 min, 1 h, and 4 h. Seventy-two enamel specimens (*n* = 72) were randomly divided into 2 groups by PLM and further by treatment groups. Then, the specimens were pre-treated with varnishes and subjected to erosive cycles (5 min, 2×/day for 4 days) by PLM. After each erosive challenge, the specimens were stored in artificial saliva. At baseline and after 4 days, the specimens were assessed for surface roughness (Ra) using 2D-surface profilometric analysis (SPA) and atomic force microscopy (AFM). Additionally, the Ca/P ratio was determined using scanning electron microscopy with energy-dispersive X-ray spectroscopy. Paired samples dependent t-test, 1-way ANOVA and 2-way ANOVA with Bonferroni post-hoc tests were used to analyse data with the level of significance set at *p* < 0.05.

**Results:**

The pH of PLM with 8% Arg-NaF was significantly higher than the other groups at 30 min and 4 h (*p* < 0.05). With paracetamol, no significant difference was observed between the baseline and post-erosive cycle measured enamel Ra (by SPA/AFM) and Ca/P ratio for all treatment groups (*p* > 0.05). The Ra determined by AFM, at the post-erosive cycle with chlorpheniramine, when treated with 4 and 8% Arg-NaF was significantly lower than the other groups (*p* < 0.05); except CPP-ACFP (*p* > 0.05). With the chlorpheniramine post-erosive cycle, the Ca/P ratio for 4, 8% Arg-NaF and CPP-ACFP treated specimens was significantly higher than the baseline Ca/P (*p* < 0.05).

**Conclusion:**

The 4%/8% Arg-NaF and MI varnish® application exhibit an enhanced preventive effect against low pH (pH < 3.0) PLM-mediated enamel erosive challenges compared to 5% NaF varnish.

## Background

The irreversible loss of tooth structure due to non-carious processes is termed tooth surface loss. Tooth surface loss usually refers to several conditions including abrasion, attrition, abfraction, and erosion. Although the term tooth surface loss is less encouraged for use in clinical situations, tooth wear or erosive tooth wear are the terms more commonly used based on the understanding that severe tooth wear rarely happens exclusively due to an individual condition and might otherwise be contributory by acidic aetiology [1]. Dental erosion is defined as the chemical loss of mineralized tooth substance caused by exposure to acids not derived from oral bacteria [2]. The prevalence of dental erosion in children ranges from 5.7 to 78%, depending on the age, causative agent, and duration of acidic exposures [3, 4]. A potential risk factor for dental erosion in children (especially medically compromised) is the consumption of paediatric liquid medicaments (PLM), which are acidic to increase vehicle stability, and high in sugar to increase palatability. The PLM are usually highly viscous, and generally consumed at a high frequency including at bedtime. Consequently, the PLM with low pH, high viscosity, and frequent consumption exhibits a high erosive potential leading to detrimental changes (erosive wear) in the enamel [5]. To prevent the deleterious enamel-erosive effect in children routinely consuming PLM, effective preventive strategies are needed besides oral hygiene measures that are routinely delivered.

Current preventive strategies against dental erosion include diet counselling, salivary glands stimulation to increase salivary flow, fluoride regimens (both, self and professionally applied), reducing intake of erosive beverages, and improving oral hygiene [6]. The effectiveness of patient-led strategies highly depends on patient compliance. However, professionally-deliverable strategies including regular topical application of sodium fluoride (NaF) and casein phosphopeptide-amorphous calcium fluoride phosphate (CPP-ACFP) varnishes can be effective as patient compliance is of a lesser concern. Both NaF and CPP-ACFP varnishes have been found to reduce enamel erosion by PLM [6]. Post-application, the varnishes form a CaF_2_-like complex on the enamel surface rendering the tissue more resistant to acid dissolution [7]. Although the current professionally-deliverable preventive strategies can counter the erosive challenges at the tissue surface, a more potent approach would modulate the PLM pH by targeting the critical aetiological element in the tooth wear process.

Arginine (Arg), a semi-essential amino acid, is regarded as a prebiotic intervention for enhanced caries prevention when delivered with F [8–11]. As a biofilm modulator, Arg is metabolised by arginolytic commensals (*Streptococcus sanguinis* and *Streptococcus gordonii*) to ammonia (NH_3_) that raises biofilm pH for ecological homeostasis [12–16]. The combined Arg-F increases enamel F uptake and enhances remineralization potential of F on incipient carious lesions [17, 18]. Due to the inherent chemical properties of Arg, it is regarded as the most basic amino acid with a high pKa of 13.80 ± 0.10 for the terminal positively-charged guanidino group [19]. When supplemented to a water-based media, Arg solubilizes to increase pH, thereby altering the media buffer potential and thus, can be a referred to as a pH modulator. Therefore, Arg can aid to counter the erosive challenges transpiring during the tooth wear process, together with the evident protective effects of F as highlighted by previous studies [20–22].

As a professionally-deliverable intervention, the caries-preventive potential of Arg-NaF varnish was explored by several studies eventually to conclude that incorporating 2% Arg in a 5% NaF varnish (Duraphat®) enhanced the anti-caries effect of Duraphat® varnish [14, 23–26]. However, no studies examining the enamel erosion preventive effect of Arg-NaF varnish were identified. As outlined, erosive tooth wear in children that routinely consume PLM is a concern, which could be alleviated with enhanced preventive measures. The Arg-NaF varnish can serve as a pH modulator while enhancing the preventive effect of F, and thus can be explored as a preventive intervention against PLM-mediated enamel erosion. Therefore, the aim of this in vitro investigation was to examine the effect of Arg-NaF varnish on preventing enamel erosion by PLM. The null hypothesis tested in the present study was that there is no difference in the enamel erosion-preventive effect of Arg-NaF, NaF, and CPP-ACFP varnishes when erosively challenged by PLM. Sequentially, the 2-tailed alternative hypothesis was that there is a significant difference in the enamel erosion-preventive effect of Arg-NaF, NaF, and CPP-ACFP varnishes when erosively challenged by PLM.

## Methods

### Study experimental design

**Fig. 1:**
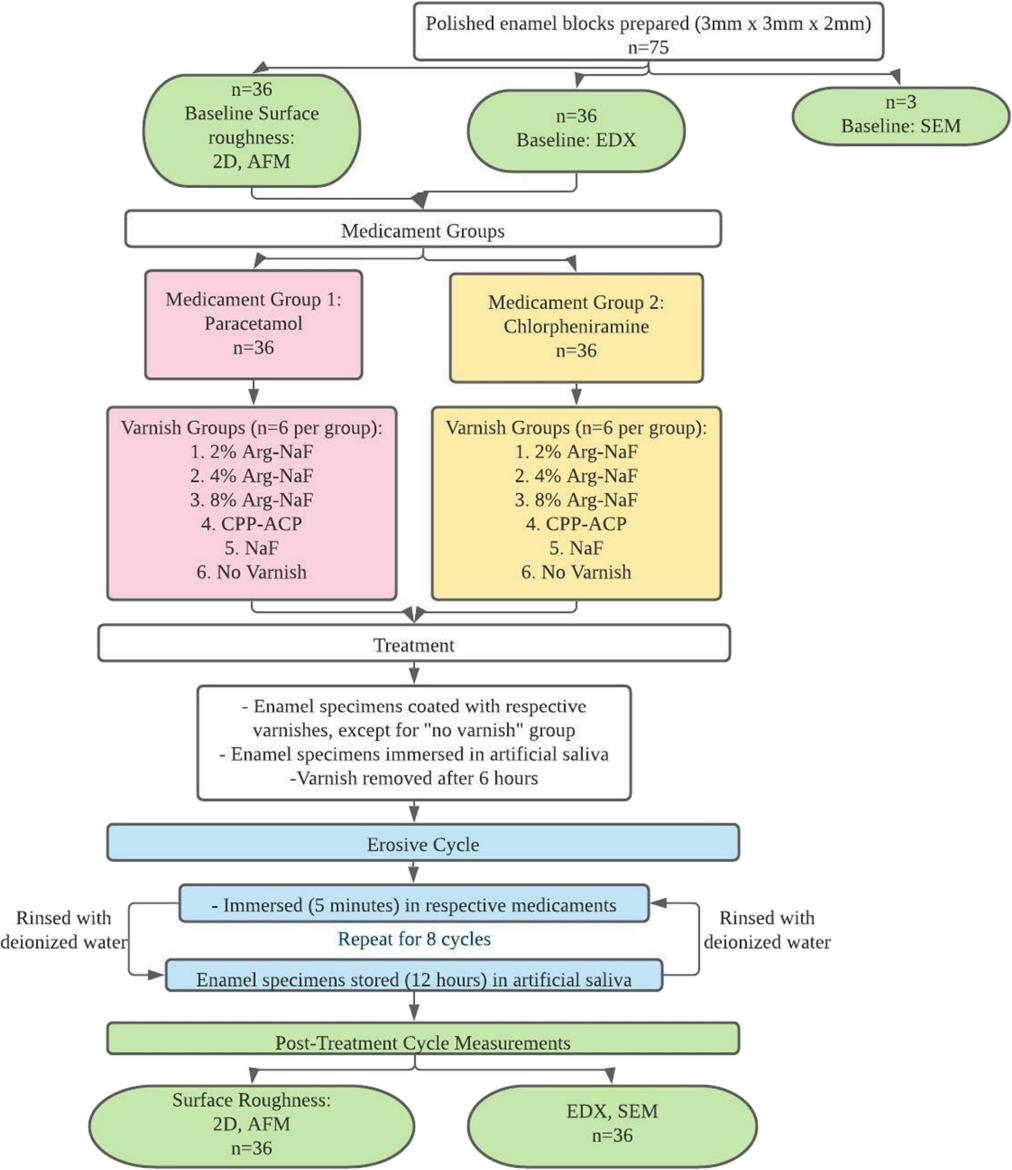
Flow chart of study experimental design

The experimental design for the present study is shown in Fig. 1. The study was approved by the Institutional Review Board (IRB) of the University of Hong Kong/Hospital Authority, Hong Kong West Cluster (IRB reference: UW 17–058).

Briefly, the erosive wear preventive effect of Arg-NaF varnishes (Arg incorporated at 2, 4, and 8% *w/v*. in Duraphat®), MI varnish® (CPP-ACFP; GC America, IL, USA), and Duraphat® varnish (5% NaF; Colgate Palmolive Company, New York, USA) was investigated on sound enamel specimens. The specimens were pre-treated with varnishes and then subjected to erosive cycles by PLM (2×/day – 5 min, each cycle) for 4 days while maintained in artificial saliva for the intervening period. After 4 days, the specimens were characterized for surface roughness using 2D-surface profilometric analysis (SPA) and atomic force microscopy (AFM). Further, the Ca/P ratio of the treated enamel surfaces was estimated by scanning electron microscopy – energy dispersive X-ray spectroscopy (SEM-EDX). To examine pre-post treatment effects, the sound specimens were also subjected to surface roughness assessment (by SPA/AFM) and Ca/P ratio determination (by SEM-EDX).

Prior to subjecting the enamel specimens for treatment with varnishes and erosive challenges with PLM, a direct pH modulatory effect of the experimental and control varnishes coated on an inert material (Perspex®) when immersed in PLM was investigated.

### Paediatric liquid medicaments (PLM)

Paracetamol and chlorpheniramine are commonly used with children to relieve pain, fever, and respiratory or allergic conditions. In the present study, PLM – Paracetamol Syrup (Paracetamol 120 mg/5 ml, Jean-Marie Pharmcal, Hong Kong) and Chlorpheniramine Maleate Syrup (Chlorpheniramine maleate 2 mg/5 ml, Jean-Marie Pharmacal, Hong Kong) were included based on the results of a previous study which concluded that the referred PLM have acidic pH [5]. For the purpose of the study, several batches of the PLM were purchased and tested under the outlined experimental conditions for technical replicates, experimental validation, and to preclude random error (if any) by PLM.

### Experimental and control treatment groups

The experimental Arg-NaF varnishes were prepared by dispensing L-arginine (A-5006, Sigma Aldrich, St. Louis, USA) at 2, 4, and 8% *w/v*. in Duraphat® (Colgate Palmolive Company, USA). Prior to the experimental application, the suspended Arg was thoroughly blended using a micro-brush to achieve solute homogeneity in the varnish solvent [23].

The control and experimental treatment groups in the present study were:

(i) 2% L-Arg in 5% NaF varnish (2% Arg-NaF)
(ii) 4% L-Arg in 5% NaF varnish (4% Arg-NaF)
(iii) 8% L-Arg in 5% NaF varnish (8% Arg-NaF)
(iv) MI varnish® - CPP-ACP with 5% NaF (CPP-ACFP)
(v) Duraphat® - 5% NaF varnish (NaF)
(vi) No varnish – negative control

### Enamel specimen preparation

Twenty extracted pristine human third molars disinfected and stored in 0.5% thymol (Sigma Aldrich, St. Louis, USA) solution at 4 °C for at least 1 week were utilised for preparation of enamel specimens. Using a microtome saw (SYD Mikki Pulley, Aichi, Japan), the enamel specimens were sectioned in the dimensions of 3 × 3 × 2 mm^3^. Prior to the specimen preparation, the molars were examined under stereomicroscope (Carl Zeiss Stereo 475,002, Oberkochen, Germany) at 8× magnification to identify enamel defects (hypoplasia, hypomineralization, fluorosis). Molars with enamel defects were excluded from the experiments. After specimen preparation, the specimens were maintained in 100% humidity until the commencement of the outlined experiments.

Before experimental cycles, the enamel specimens were embedded in resin cement (RelyX Unicem, 3 M ESPE, St. Paul, USA) with the enamel surface exposed. Then, the resin-enamel surfaces were sequentially polished with silicon carbide paper of 220, 600, 1000, 2000, and 4000 grit (Buehler, Lake Bluff, IL, USA). The specimens were cleaned in an ultrasonic cleaner (Wise Clean WUC-D10H, Daihan, Seoul, South Korea) for 5 mins while immersed in deionized water (DIW) prior to being subjected to baseline characterizations.

### Artificial saliva preparation

The artificial saliva was freshly prepared by adding CaCl_2_, MgCl_2_.6H_2_O, KH_2_PO_4_, NaN_3_, KCl, HEPES buffer, and phenol red solution (Sigma Aldrich, St. Louis, USA) to DIW as per a previous study [24]. The pH of the solution was adjusted to 7.0 using 5 M KOH. Prior to use, the solutions were thoroughly mixed by agitating the solution jar.

### pH measurements of PLM

The pH of the PLM was measured against external standards with pH: 4.01, 7.0, 10.01 using a pH electrode (Orion, Thermo Scientific, UK, NE) attached to a benchtop potentiometer (Oakton Ion 2700, OAKTON Instruments, IL., USA). Initially, the baseline pH of the PLM was measured without any intervention. Then, the Perspex® blocks were coated with respective varnishes and introduced in Sterilin™ tubes (Bijou, Thermo Scientific, Newport, UK) with PLM to measure pH at 0 min, 30 min, 1 h and 4 h. In between measurements, the tubes were left static on the laboratory bench at room temperature.

### Treatment and erosive cycles

A single trained examiner applied a thin layer of varnish to the enamel specimens randomly distributed to the respective groups, primarily based on PLM and experimental/control treatment. The varnish coated specimens were then immersed in 15 ml of artificial saliva for 6 h. Then, the varnish was gently removed from the specimens with the help of a scalpel blade, and the specimens were rinsed in DIW and dried using fibreless laboratory napkins (Kimwipes, Kimberly-Clark Professional, TX, USA). Erosive challenge by the PLM then commenced. The specimens were subjected to 5 min of erosive attack by the respective PLM every 12 h, for 4 days. After each erosive attack, the specimens were rinsed with DIW, dried, and suspended in freshly prepared artificial saliva. After the last erosive challenge, the specimens were stored in 15 ml of artificial saliva for 24 h, before being removed for post-erosive cycle characterizations. While awaiting characterizations, the specimens were stored in a 100% moist environment assembled using 0.5% thymol solution.

### Surface profilometry analysis (SPA)

Using the 2D surface profiler (Surtronic 3+ surface profiler, AMETEK Taylor Hobson company, LC, UK), the enamel specimens were measured for surface roughness (Ra) in μm using the stylus, with the cut-off length set at 0.25 mm. Randomly, 3 surface profilometric readings were taken per specimen, at each baseline and post-treatment/erosive cycle.

### Atomic force microscopy (AFM)

The specimens were scanned using AFM (Model Dimension Edge, Bruker, MA, USA) to determine Ra in μm as per a previous study [27]. Three different interest areas (30 × 30 μm^2^) were randomly selected from each specimen to scan height profiles with an AFM probe (Model OTESPA-R3, Bruker, MA, USA) to eventually analyse Ra with the AFM software (NanoScope Analysis, v. 1.50, Bruker, MA, USA). The mean of obtained Ra was computed for each specimen for further analysis as per the respective groups.

### Scanning electron microscopy with energy-dispersive X-ray spectroscopy (SEM-EDX)

The enamel specimens were examined by EDX (Model 550i, IXRF Systems, TX, USA) using accelerating voltage of 15 kV, at baseline and after treatment/erosive cycles. The Ca/P ratio (wt.%) was determined at 500× magnification with SEM (SU1510, Hitachi, Ibaraki, Japan) unit by scanning 3 randomly identified areas on the surface of interest. The mean Ca/P ratio for each specimen was then calculated for further statistical analysis.

The treated specimens (post-erosive cycle) were then imaged using SEM (Hitachi SU1510, Japan) at three random areas on the exposed enamel surface at 500× magnification. For the purpose of imaging, the specimens were first sputtered with platinum (80%)–palladium (20%) and then examined for surface topography. For baseline qualitative assessment, sound enamel specimens (*n* = 3) were imaged after platinum-palladium sputtering. Any ultramicroscopic alterations associated with erosive wear were qualitatively assessed in comparison to the SEM images obtained for sound enamel specimens.

### Statistical analysis

The quantitative data obtained in the present study was entered in MS Excel spreadsheet (Microsoft, WA, USA) for further statistical analysis using SPSS v. 26 (IBM statistics, IL, USA).

Paired samples dependent t-test was used to analyse statistically significant differences (if any) between the: 1) PLM pH (at baseline) and the pH of the PLM after inclusion of Perspex® coated varnishes; 2) Ra at baseline and post-erosive cycles by SPA/AFM; and 3) Ca/P ratio at baseline and post-erosive cycles for all treatment groups challenged by either of the PLM.

One-way ANOVA with Bonferroni post-hoc test was performed to identify differences between the treatment groups at each baseline and post-erosive cycle for characterizations of Ra (by SPA/AFM) and Ca/P ratio by SEM-EDX with relevant computed mean difference (modulus of the difference between baseline and post-erosive cycle determined variable).

Two-way ANOVA with Bonferroni post-hoc test (at all levels) was used to analyse the effect of Factor 1: treatment groups; and Factor 2: time points (0 min, 30 min, 1 h, and 4 h) on the determined pH of the PLM. Further, factor interaction analysis was undertaken to estimate interaction-based changes on the PLM pH determined with respective treatment groups and different time-points.

For all statistical tests, the level of significance was set at *p* < 0.05.

## Results

### Medicament pH measurements

The results of the measured pH (mean ± SD) for PLM at baseline and post-immersion of Perspex® coated varnishes (at time intervals – 0 min, 30 min, 1 h and 4 h) in PLM are presented in Table 1. With paracetamol, pH of the PLM with 8% Arg-NaF and CPP-ACFP intervention group (at 0 min) was significantly higher than the baseline pH measured for the PLM (*p* < 0.05). While for chlorpheniramine, except for the negative and NaF control, the pH with all treatment groups (at 0 min) was significantly higher than the measured pH of PLM at baseline (*p* < 0.05).

**Table 1:** pH (Mean ± SD) of PLM at baseline (without Perspex blocks) and at 0 min, 30 mins, 1 h, and 4 h after immersing Perspex blocks coated with varnishes: A) Paracetamol; and B) Chlorpheniramine

**A) Paracetamol**					
**Groups**	**pH of PLM** **(Mean ± SD)**	**pH of PLM after immersing Perspex blocks coated with varnishes** **(Mean ± SD)**			
		**0 min**	**30 mins**	**1 h**	**4 h**
**2% Arg-NaF**	4.68 ± 0.08^A^	4.69 ± 0.06^Aa^	4.65 ± 0.05^Aa^	4.63 ± 0.04^Aa^	4.66 ± 0.04^Aa^
**4% Arg-NaF**	4.66 ± 0.07^A^	4.69 ± 0.05^Aa^	4.66 ± 0.05^Aa^	4.64 ± 0.04^Aa^	4.65 ± 0.04^Aa^
**8% Arg-NaF**	4.65 ± 0.06^A**^	5.0 ± 0.40^Ba^	4.78 ± 0.05^Ba^	4.75 ± 0.07^Ab^	4.81 ± 0.19^Ba^
**CPP-ACFP**	4.66 ± 0.06^A**^	5.30 ± 0.22^Ca^	4.63 ± 0.03^Ab^	4.62 ± 0.04^Ab^	4.62 ± 0.03^Ab^
**NaF**	4.66 ± 0.06^A^	4.67 ± 0.06^Aa^	4.64 ± 0.03^Aa^	4.61 ± 0.05^Aa^	4.61 ± 0.05^Aa^
**No varnish**	4.66 ± 0.06^A^	4.68 ± 0.02^Aa^	4.64 ± 0.03^Aa^	4.62 ± 0.04^Aa^	4.63 ± 0.04^Aa^
**B) Chlorpheniramine**					
**Groups**	**pH of PLM** **(Mean ± SD)**	**pH of PLM after immersing Perspex blocks coated with varnishes** **(Mean ± SD)**			
		**0 min**	**30 mins**	**1 h**	**4 h**
**2% Arg-NaF**	2.55 ± 0.02^A**^	2.62 ± 0.05^Ba^	2.54 ± 0.04^Ab^	2.53 ± 0.01^Ab^	2.55 ± 0.02^Ab^
**4% Arg-NaF**	2.56 ± 0.02^A**^	2.64 ± 0.05^Ba^	2.61 ± 0.05^Ab^	2.59 ± 0.05^Ab^	2.56 ± 0.06^Ab^
**8% Arg-NaF**	2.56 ± 0.03^A**^	2.61 ± 0.02^Aa^	2.68 ± 0.07^Bb^	2.67 ± 0.11^Bb^	2.68 ± 0.13^Bb^
**CPP-ACFP**	2.54 ± 0.03^A**^	2.65 ± 0.07^Ba^	2.67 ± 0.10^Ba^	2.70 ± 0.09^Ba^	2.72 ± 0.09^Ba^
**NaF**	2.55 ± 0.03^A^	2.60 ± 0.10^Aa^	2.58 ± 0.06^Aa^	2.56 ± 0.05^Aa^	2.56 ± 0.03^Aa^
**No varnish**	2.54 ± 0.04^A^	2.54 ± 0.04^Aa^	2.53 ± 0.03^Aa^	2.53 ± 0.02^Aa^	2.52 ± 0.01^Aa^

Paired t-tests were done to check for any differences in PLM pH levels at baseline and after Perspex blocks with varnishes were added at 0 min

Two-way ANOVA was used to check the effect of factors (Factor 1: varnishes and Factor 2: time interval) on measured pH and further the effect of the factor interaction on pH. (Note: We did not include the pH of PLM at baseline in the 2-way ANOVA test as the PLM at baseline had no Perspex block coated with varnishes)

Different superscript uppercase and lowercase letters represent differences (*p* < 0.05) in each column and row, respectively with Bonferroni post-hoc text

* Paired t-test (PLM v/s. 0 min), **p < 0.05

The results of 2-way ANOVA showed that the effect of both factors (Factor 1 & 2) and their interaction on the determined pH was significantly different (*p* < 0.001). For paracetamol PLM, at 0 min, the pH of 8% Arg-NaF and CPP-ACFP was significantly higher than the other groups (*p* < 0.05). Furthermore, the pH measured at 30 min and 4 h for 8% Arg-NaF was significantly higher than the other groups (*p* < 0.05). The pH at 1 h for 8% Arg-NaF group was significantly lower than the pH at other time points (*p* < 0.05), while the pH measured with CPP-ACFP at 0 min was higher than the pH at 30 min, 1 h, and 4 h (*p* < 0.05).

For chlorpheniramine, at 0 min, the pH with 2, 4% Arg-NaF, and CPP-ACFP was significantly higher than the other groups (*p* < 0.05). Consistently, at 30 min, 1 h and 4 h, the measured pH with 8% Arg-NaF and CPP-ACFP was significantly higher than the other groups (*p* < 0.05). Furthermore, for all Arg-NaF groups, the pH at 0 min was significantly higher than the pH at 30 min, 1 and 4 h (*p* < 0.05), while the pH at all time-points for the other groups were similar with no statistically significant difference (*p* > 0.05).

Therefore, a significant increase in pH of PLM for Perspex®-coated varnishes with 8% Arg-NaF was evident at 30 min and 4 h, post-immersion.

### Surface roughness assessment

The data for Ra (in μm) measured with SPA and AFM are shown in Tables 2 and 3, respectively. With paracetamol (Tables 2 & 3), no significant differences could be identified between the mean Ra for all groups at baseline and post-erosive cycle (*p* > 0.05); and additionally, between the baseline and post-erosive cycle with all groups (*p* > 0.05).

**Table 2:** Surface roughness (Mean ± SD) of enamel specimens (in μm) determined by 2D-SPA at baseline and post-erosive cycle

Groups	Paracetamol			Chlorpheniramine		
	Baseline	Post-Erosive Cycle	Mean Difference (Post-Erosive Cycle-Baseline)	Baseline	Post-Erosive Cycle	Mean Difference (Post-Erosive Cycle-Baseline)
	Mean ± SD	Mean ± SD		Mean ± SD	Mean ± SD	
**2% Arg-NaF**	0.09 ± 0.01^Aa^	0.10 ± 0.04^Aa^	0.02^A^	0.09 ± 0.01^Aa^	0.29 ± 0.07^Ab^	0.20^A^
**4% Arg-NaF**	0.08 ± 0.02^Aa^	0.10 ± 0.02^Aa^	0.02^A^	0.08 ± 0.02^Aa^	0.28 ± 0.10^Ab^	0.20^A^
**8% Arg-NaF**	0.08 ± 0.01^Aa^	0.14 ± 0.09^Aa^	0.07^A^	0.09 ± 0.02^Aa^	0.25 ± 0.11^Ab^	0.16^A^
**CPP-ACFP**	0.08 ± 0.01^Aa^	0.12 ± 0.05^Aa^	0.04^A^	0.08 ± 0.01^Aa^	0.23 ± 0.08^Ab^	0.15^A^
**NaF**	0.09 ± 0.02^Aa^	0.09 ± 0.02^Aa^	0.004^A^	0.10 ± 0.01^Aa^	0.27 ± 0.08^Ab^	0.17^A^
**No varnish**	0.07 ± 0.01^Aa^	0.10 ± 0.03^Aa^	0.02^A^	0.10 ± 0.02^Aa^	0.46 ± 0.17^Bb^	0.37^B^

Superscript uppercase letters represent differences (*p* < 0.05) in each column as analysed using 1-way ANOVA and Bonferroni post-hoc test

Superscript lowercase letters represent differences (*p* < 0.05) in each row as analysed using a dependent t-test

**Table 3:** Surface roughness (Mean ± SD) of enamel specimens (in μm) determined by AFM at baseline and post-erosive cycle

Groups	Paracetamol			Chlorpheniramine		
	Baseline	Post-Erosive Cycle	Mean Difference (Post-Erosive Cycle-Baseline)	Baseline	Post-Erosive Cycle	Mean Difference (Post-Erosive Cycle-Baseline)
	Mean ± SD	Mean ± SD		Mean ± SD	Mean ± SD	
**2% Arg-NaF**	0.01 ± 0.002^Aa^	0.01 ± 0.003^Aa^	0.001^A^	0.01 ± 0.003^Aa^	0.10 ± 0.01^ACb^	0.09^AC^
**4% Arg-NaF**	0.01 ± 0.002^Aa^	0.01 ± 0.002^Aa^	0.000^A^	0.01 ± 0.002^Aa^	0.06 ± 0.02^Bb^	0.05^B^
**8% Arg-NaF**	0.01 ± 0.001^Aa^	0.01 ± 0.004^Aa^	-0.001^A^	0.01 ± 0.002^Aa^	0.06 ± 0.02^Bb^	0.04^B^
**CPP-ACFP**	0.01 ± 0.001^Aa^	0.01 ± 0.002^Aa^	0.001^A^	0.01 ± 0.003^Aa^	0.07 ± 0.01^ABb^	0.06^AB^
**NaF**	0.01 ± 0.001^Aa^	0.01 ± 0.004^Aa^	0.000^A^	0.01 ± 0.002^Aa^	0.11 ± 0.03^Cb^	0.09^C^
**No varnish**	0.01 ± 0.001^Aa^	0.01 ± 0.002^Aa^	-0.001^A^	0.01 ± 0.002^Aa^	0.13 ± 0.03^Cb^	0.12^C^

Superscript uppercase letters represent differences (*p* < 0.05) in each column as analysed using 1-way ANOVA and Bonferroni post-hoc test

Superscript lowercase letters represent differences (*p* < 0.05) in each row as analysed using a dependent t-test

For chlorpheniramine, the mean Ra measured by SPA (Table 2) at the post-erosive cycle was significantly lower for all the intervention groups compared to the negative control (*p* < 0.05). Conversely, significant differences were observed in the Ra determined using AFM (Table 3) between the treatment groups at the post-erosive cycle as 4%/8% Arg-NaF = CPP-ACFP < NaF < No varnish (*p* < 0.05); 2% Arg-NaF = CPP-ACFP (*p* > 0.05) and 2% Arg-NaF = NaF control (*p* > 0.05). Irrespective of the characterization technique, the Ra measured at the post-erosive cycle (Tables 2 & 3) was significantly higher than the baseline for all the groups (*p* < 0.05).

Although the Ra determined for paracetamol did not reveal significant differences between baseline and post-erosive cycle for all treatment groups; the Ra determined by AFM, at the post-erosive cycle with chlorpheniramine, when treated with 4 and 8% Arg-NaF was significantly lower than the other groups (*p* < 0.05); except CPP-ACFP (*p* > 0.05).

### Ca/P ratio by EDX

The Ca/P ratio determined at baseline and post-erosive cycle is presented in Table 4. For paracetamol, no significant differences could be identified between and within the groups at baseline and post-erosive cycle (*p* > 0.05). With chlorpheniramine challenge, the Ca/P ratio with 4%/8% Arg-NaF and CPP-ACFP was significantly higher at the post-erosive cycle than the baseline (*p* > 0.05); while for the negative control contrarily the Ca/P ratio decreased significantly compared to the baseline (*p* < 0.05). For the other groups, no significant difference could be identified between the baseline and post-erosive cycle Ca/P ratio (*p* > 0.05). Furthermore, the Ca/P ratio at the post-erosive cycle for CPP-ACFP was significantly higher than the NaF and no varnish controls (*p* < 0.05), while there was no difference in Ca/P between 2%/4%/8% Arg-NaF and CPP-ACFP (*p* > 0.05) and then similarly between 2%/4%/8% Arg-NaF and NaF (*p* > 0.05).

**Table 4:** Ca/P ratio (Mean ± SD) of enamel specimens determined using SEM-EDX at baseline and post-erosive cycle

Groups	Paracetamol			Chlorpheniramine		
	Baseline	Post-Erosive Cycle	Mean Difference (Post-Erosive Cycle-Baseline)	Baseline	Post-Erosive Cycle	Mean Difference (Post-Erosive Cycle-Baseline)
	Mean ± SD	Mean ± SD		Mean ± SD	Mean ± SD	
**2% Arg-NaF**	1.63 ± 0.07^Aa^	1.65 ± 0.09^Aa^	0.02^A^	1.56 ± 0.03^Aa^	1.60 ± 0.07^ABCa^	0.04^AB^
**4% Arg-NaF**	1.63 ± 0.04^Aa^	1.65 ± 0.03^Aa^	0.02^A^	1.57 ± 0.03^Aa^	1.63 ± 0.06^ABb^	0.06^AB^
**8% Arg-NaF**	1.62 ± 0.04^Aa^	1.64 ± 0.06^Aa^	0.03^A^	1.53 ± 0.04^Aa^	1.62 ± 0.06^ABb^	0.08^A^
**CPP-ACFP**	1.62 ± 0.04^Aa^	1.68 ± 0.12^Aa^	0.06^A^	1.54 ± 0.05^Aa^	1.68 ± 0.07^Bb^	0.14^A^
**NaF**	1.61 ± 0.02^Aa^	1.61 ± 0.11^Aa^	0.01^A^	1.54 ± 0.05^Aa^	1.57 ± 0.08^ACa^	0.02^AB^
**No varnish**	1.63 ± 0.07^Aa^	1.59 ± 0.06^Aa^	-0.07^A^	1.56 ± 0.03^Aa^	1.51 ± 0.03^Cb^	-0.05^B^

Superscript uppercase letters represent differences (*p* < 0.05) in each column as analysed using 1-way ANOVA and Bonferroni post-hoc test

Superscript lowercase letters represent differences (*p* < 0.05) in each row as analysed using a dependent t-test

Briefly, for the paracetamol group, no significant difference was observed between the baseline and post-erosive cycle Ca/P ratio for all treatment groups (*p* > 0.05). However, with chlorpheniramine post-erosive cycle, the Ca/P ratio for 4, 8% Arg-NaF and CPP-ACFP treated specimens was significantly higher than the baseline (*p* < 0.05) and for CPP-ACFP, the Ca/P at post-erosive cycle was higher than NaF and negative control (*p* < 0.05).

### Imaging by SEM

**Fig. 2:**
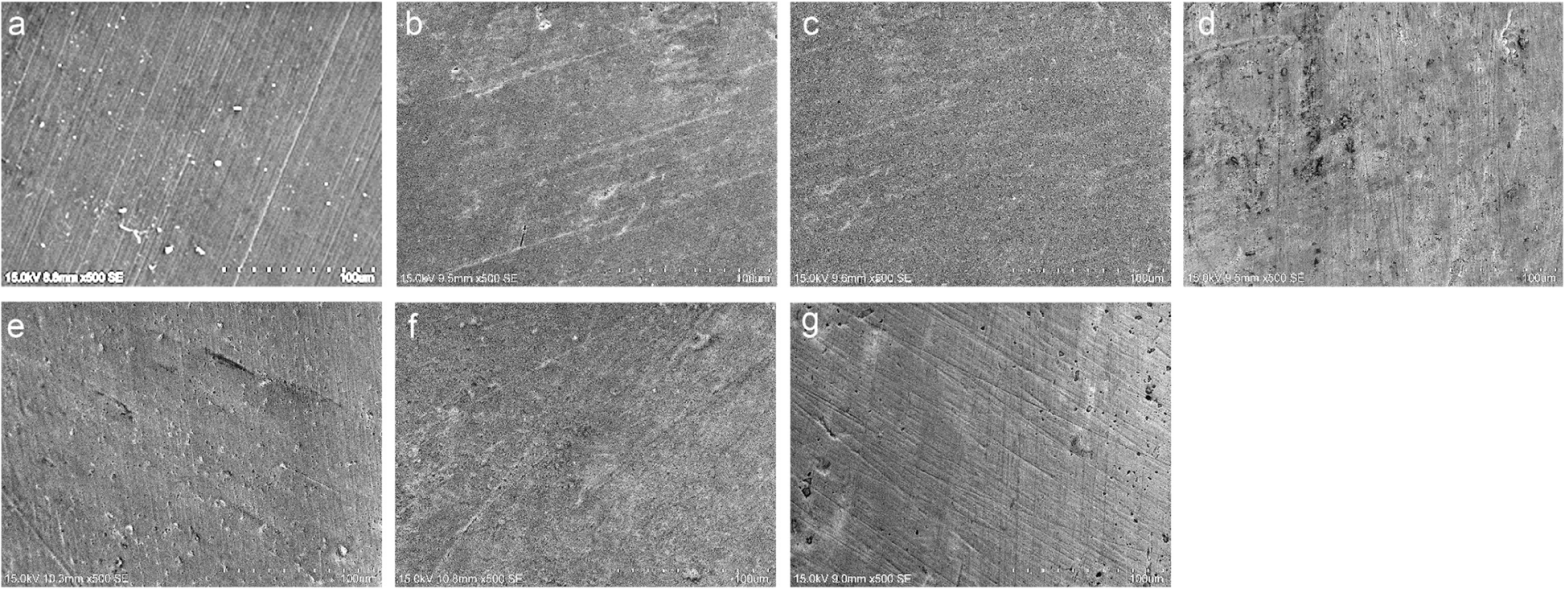
Baseline and post-erosive cycle SEM images of enamel specimens (at 500×) when challenged with Paracetamol. SEM images at **a** baseline; and post-erosive cycle in **b** 2% Arg-NaF; **c** 4% Arg-NaF; **d** 8% Arg-NaF; **e** CPP-ACFP; **f** NaF; **g** No varnish groups

**Fig. 3:**
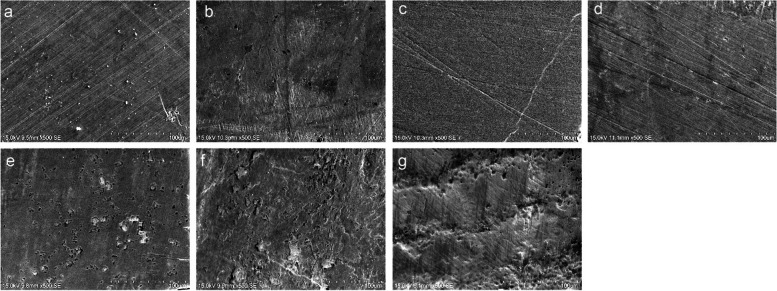
Baseline and post-erosive cycle SEM images of enamel specimens (at 500×) when challenged with Chlorpheniramine. SEM images at **a** baseline; and post-erosive cycle in **b** 2% Arg-NaF; **c** 4% Arg-NaF; **d** 8% Arg-NaF; **e** CPP-ACFP; **f** NaF; **g** No varnish groups

Figures 2 and 3 show the SEM images of sound enamel and enamel specimens erosively challenged by PLM paracetamol and chlorpheniramine, respectively. Similar to the results of Ra and Ca/P ratio, no characteristic differences could be observed between the baseline and post-erosive cycle specimens when erosively challenged by paracetamol (Fig. 2). Whereas, for specimens challenged by chlorpheniramine, the post-erosive cycle images of 4%/8% Arg-NaF treatment groups **(**Fig. 3cd) appeared smooth and similar to the baseline image **(**Fig. 3a), but contrasted markedly with the post-erosive cycle SEM image of NaF **(**Fig. 3f) and no varnish groups **(**Fig. 3g**)**, which showed generalized irregular depressions and areas of erosion that coalesced. Concurrently, the post-erosive cycle SEM image of 2% Arg-NaF **(**Fig. 3b) and CPP-ACFP **(**Fig. 3e) showed more distinct and smaller areas of pitting compared to NaF **(**Fig. 3f) and no varnish controls **(**Fig. 3g).

In summary, the results of the present study suggest that 4%/8% Arg-NaF and CPP-ACFP impart an enhanced preventive effect against erosive attacks by chlorpheniramine (pH < 3.0); while the interventions do not provide an additive preventive effect to enamel specimens challenged by paracetamol (pH > 4.50).

## Discussion

In the present study, we aimed to examine the enamel erosion preventive effect of the experimental Arg-NaF varnishes (at 2, 4, and 8% *w/v*.) when erosively challenged by PLM (paracetamol and chlorpheniramine) compared to the control NaF and CPP-ACFP varnishes, which are known to impart a preventive effect against enamel erosion by PLM [6]. The results of the study confirm that incorporating 4%/8% Arg in NaF varnish and the commercial control CPP-ACFP provide an enhanced preventive effect against the erosive challenge by PLM with pH < 3.0 (chlorpheniramine). However, when the pH of the PLM is greater than 4.50 (paracetamol), the preventive effect imparted by the interventions were similar to the controls. The results were based on the data obtained using characterizations like PLM pH measurements, Ra determination (in μm) using SPA/AFM and Ca/P estimation (wt.%) by SEM-EDX. Therefore, based on the results of the present study, the null hypothesis that there is no difference between the enamel erosion preventive effect of Arg-NaF, NaF, and CPP-ACFP varnishes is rejected and the alternative hypothesis is accepted which is limited to the erosive wear by PLM with low pH (*p* < 3.0).

Frequently consumed by children, paracetamol and chlorpheniramine, were used for PLM-mediated erosive challenges in the present study as pH of these PLM were 4.74 and 2.50, respectively. A severe erosive pattern was observed with chlorpheniramine, primarily due to the pH of the medicament. Furthermore, chlorpheniramine maleate is the active ingredient of the Chlorpheniramine Maleate Syrup (used in this study) which when dispensed in the oral environment dissociates to form a chlorpheniramine base and maleic acid. The acid component chemisorbs to the hydroxyapatite (HA) through ionic interactions [28] and has been previously investigated for its use as an etchant [29]. Conversely, the erosive effect of paracetamol was less detrimental, despite the fact that the pH being < 5.0 which is lesser than the critical pH, that imparts enamel surface and sub-surface changes through continuous presence of biofilm-mediated bacterial acids in high caries-risk patients [30]. Therefore, PLM with pH > 4.50 might not result in obvious erosive wear changes when routine oral hygiene and professionally-deliverable preventive measures are regularly exercised.

The CPP-ACFP (as MI varnish®) has been available in the market for several years and is known to reduce surface roughness caused by dental erosion [6]. In addition, MI varnish® increases F uptake along with precipitation of Ca and phosphates (PO_4_^3−^) to subsurface enamel incipient carious-lesions, thereby substantially enhancing remineralization [31, 32]. The casein in CPP-ACFP modifies enamel mechanical properties, rendering it less susceptible to mineral loss with acidic/erosive challenges [32]. Incorporating Arg in NaF varnish (Duraphat®) also increases the enamel F uptake and enhances the remineralization potential of Duraphat® by forming Arg-Ca-F_2_ complexes which could additionally contribute to enamel resistance against erosive challenges by PLM [18, 24, 26]. Furthermore, enamel Arg uptake and subsequent release during erosive challenges might demonstrate a pH modulatory potential of the basic amino acid [26].

The erosivity of PLM depends on several factors including the content pKa, temperature, viscosity, and the consumption frequency. As reported earlier, acidic PLM can result in softening of enamel and increase the risk of erosive wear [33], which is further dependent on tooth-related factors such as the tooth mineral content, possibility of PLM reactivity, and enamel resistance against low pH conditions. The amino acid Arg used as an interventional element to prepare experimental varnishes (Arg-NaF) in the present study can impart a pH modulatory effect at high concentrations which can be delineated from the results of pH determination. The results of a previous study suggested that incorporating higher concentrations of Arg (at 8% *w/v*.) in Duraphat® influences the chemical properties of the varnish leading to a higher Arg release which is dependent on the molecular dynamics initiating a higher order energy due to the interaction between Arg and F, when the concentrations of Arg are greater than F [23]. Therefore, during the experimental phases of the present study, higher release of Arg with 8% Arg-NaF led to pH changes challenging the buffer potential of PLM being the most basic amino acid with pKa = 13.80 for the terminal guanidino group.

Based on the overall results of PLM-mediated erosive challenges, it can be explicitly deciphered that the varnish treatment groups exhibited an order of protection against erosive wear when challenged with PLM containing chlorpheniramine (pH < 3.0) as opposed to paracetamol (pH > 4.50) whereby all treatment groups demonstrated no additive preventive effect. Therefore, forthcoming, the results with chlorpheniramine-mediated erosive challenges and the subsequent effect of varnish applications are discussed. With SPA, the measured Ra for all treatment groups was significantly lower than the negative control. However, the results with AFM identified significant differences between the treatment groups. This is likely as the 2D surface profiler runs across a straight line while detecting limited surface characteristics roughness. Whereas, the AFM scans surface area and detects finer details while characterising the surface of interest and thus, such differences between characterization were identified.

The results of Ca/P determined using SEM-EDX align well with the results of Ra estimated with AFM whereby 4%/8% Arg-NaF and CPP-ACFP had significantly higher Ca/P ratio than the baseline and the negative control. The higher Ca/P ratio than the baseline could be attributed to the precipitation of Ca and PO_4_^3−^ by varnish treatment and subsequent interaction with the components of artificial saliva. However, the difference from the negative control could be due to the presence of complexes formed by Arg, Ca, F, PO_4_^3−^ for Arg-NaF and Ca, F, PO_4_^3^ for CPP-ACFP which resisted the erosive challenges by acidic PLM. Furthermore, the SEM results qualitatively explain that post-erosive cycle the treatment with 4%/8% Arg-NaF and CPP-ACFP exhibited similar surface characteristics with minimal differences (if any) to that of baseline which is in accordance with the results of determined Ra and Ca/P ratio with the Ca/P closest to HA i.e. 1.67.

The results of the present study suggest that incorporating Arg at 4%/8% *w/v*. in Duraphat® enhances the erosive wear preventive potential of Duraphat® similar to CPP-ACFP. In a previous study on Arg-NaF varnish, the study contributors raised concerns on incorporating 8% Arg in Duraphat® which exhibited cytotoxic effects on HGF-1. Use of 8% Arg-NaF was recommended against as it leads to uncontrolled F release with excipients from the varnish matrix, and hence can be detrimental to gingival fibroblasts [26]. It was further suggested that F varnish preparations that lead to short-term rapid F release and subsequent decline in F release potential do not demonstrate promising results [34]. With 4% Arg-NaF demonstrating an erosion preventive potential similar to CPP-ACFP, it can be an enhanced intervention to prevent erosive wear in patients continuously exposed to acidic PLM. In addition, another study concluded that 4% Arg-NaF enhances the biofilm modulatory effect of Duraphat® through release of Arg and F as varnish active elutes [14]. Therefore, incorporating 4% Arg in Duraphat® might be promising for both biofilm homeostasis and erosive wear prevention via the pH modulation potential.

Although the study was comprehensively performed to address the objectives, the study limitations include being an in vitro investigation, experiments on third permanent molars due to difficulty in obtaining sound primary teeth, and teeth-related characterizations limited to SPA, AFM, and SEM-EDX. However, the results of the study present a platform for further investigation of the 4%/8% Arg-NaF varnish as a professionally-deliverable erosive wear preventive agent, which can be applied to patients with suspected or proven milk protein allergy whereby application of MI varnish® is contra-indicated. To delineate the erosive wear preventive potential of Arg-NaF varnish on a larger scale, future research targeted on investigating the erosive wear preventive effect of Arg-NaF varnish against the regularly consumed acidic fruit juices and soft drinks can provide additional insights.

## Conclusion

Under the conditions of the present study, it can be concluded that –

1. The 4%/8% Arg-NaF and CPP-ACFP demonstrated an enhanced preventive effect against the erosive challenge by PLM with pH < 3.0 (chlorpheniramine) when compared to NaF.
2. No additive preventive effect was shown by the tested varnishes when PLM with pH > 4.50 (paracetamol) was used, as the PLM did not substantially contribute to erosive wear.

Therefore, it can be inferred that the 4%/8% Arg-NaF and MI varnish® application exhibit an enhanced preventive effect against low pH (pH < 3.0) PLM-mediated enamel erosive challenges compared to 5% NaF varnish.

## Data Availability

The datasets used and/or analyzed during the current study are available from the corresponding author on reasonable request.
